# Study of Lateral Displacements and the Natural Frequency of a Pedestrian Bridge Using Low-Cost Cameras

**DOI:** 10.3390/s20113217

**Published:** 2020-06-05

**Authors:** Yiannis Fradelos, Olga Thalla, Irene Biliani, Stathis Stiros

**Affiliations:** Department of Civil Engineering, Patras University, 26500 Patras, Greece; yiannisfrad@gmail.com (Y.F.); olgath199355@gmail.com (O.T.)

**Keywords:** bridge, structural health monitoring, vision, dynamic displacements, modal frequency

## Abstract

Vision-based techniques are frequently used to compute the dynamic deflections of bridges but they are rather computationally complicated and require demanding instrumentation. In this article, we show that it is possible to reconstruct the 2-D kinematics of flexible bridges using a simplified algorithm to analyze common video imagery. The only requirements are that the movement of the control points is clearly visible on the images and that next to each control point, there exist vertical and horizontal bridge elements defining the image scale. We applied this technique during controlled, forced excitations of a timber bridge that was stiff in the vertical but very flexible in the lateral axis because of cumulated damage. We used videos from low-cost cameras, in which the changes of the pixel coordinates of several control points during excitation events and their attenuation were clear. These videos were obtained during two annual structural health monitoring surveys using numerous sensors (Global Navigation Satellite Systems (GNSS), robotic total station (RTS), accelerometers), and hence the output of the video analysis was fully controlled. Because of various errors, the transformation of the video image coordinates into bridge coordinates yielded spurious deflections along the main axis of the bridge, which were used to control the uncertainty of our results. We found that the computed lateral deflections (i) were statistically significant, (ii) satisfied structural constraints, and (iii) were consistent with structural estimates derived from other sensors. Additionally, they provided accurate estimates of the natural frequency and the damping factor of the bridge. This approach can be applied in other cases of monitoring of flexible structures if the requirements for planar deformation, pixel resolution and scale definition are satisfied.

## 1. Introduction

Vision-based techniques have been extensively used in the monitoring of static and dynamic deformations of various structures [1], especially of bridges (e.g., Xu and Brownjohn [2]). The overall approach is based on the exploitation of a linear relationship between the 3-D reference (“world”) coordinate system of a control point on the bridge with its 2-D coordinates deduced from a series of images; the latter can be obtained using a wide range of image-recording hardware, from common video-recording cameras to high-rate, high-resolution cameras and CCD cameras. This relationship is in the form:

(1) $${\left[{\;x}_{p},{\;y}_{p},1\right]}^{T}=\;k\;A\;T\;{\left[x_{r},{\;y}_{r},{\;z}_{r},1\right]}^{T},\;$$

where p and r indicate the image (photo) and reference coordinate systems, respectively; k is a scale factor; A is a 3 × 3 matrix describing the camera characteristics; and T is a 4 × 3 matrix defining the rotation and translation of the image relative to the reference coordinate system (see References [3,4]). The details of Equation (1) are given in the Appendix A.

Equation (1) contains several variables and unknown parameters and for this reason, its analysis requires high-quality instrumentation, the calibration of observations, and rather complicated analysis that may lead to the identification of displacements at a subpixel scale [5], even of microtremors [6], and can hence permit full-scale structural identification (including the modeling of all modes of a structure) [7].

However, in some cases, the overall vision-based analysis process can be highly simplified. This is especially possible if the deformation is planar [8], if it is possible to introduce constraints in the bridge movement, if the camera location is stable and in a known position and orientation, and especially if the displacement signal is unambiguously captured by the images at a multipixel-per-mm level. These conditions highly simplify the analysis algorithm and permit modeling of the dynamic deformation of a bridge using simple computational techniques and low-cost sensors (cameras).

**Figure 1:**
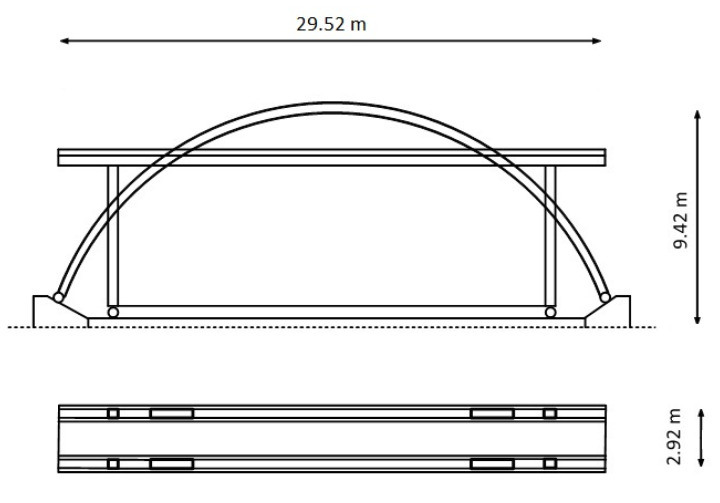
Geometric characteristics of the Kanellopoulos timber bridge (Patras, Greece). Top: side view, bottom: plan view.

**Figure 2:**
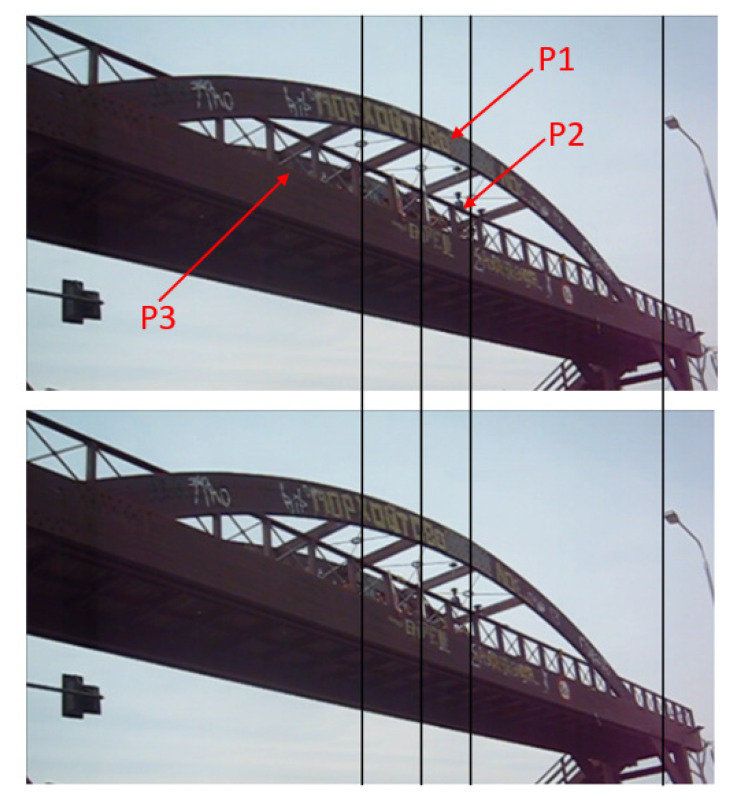
Two snapshots of a part of the Kanellopoulos bridge during a forced controlled excitation. Vertical lines are added to highlight the high amplitude of the bridge deflections. In the lower figure, the polygonal connections of steel X-braces at the roof of the bridge are seen to be displaced during a force-excitation event by jumping pedestrians (hardly shown on the bridge). The width of the polygonal connection, about 15 cm, indicates the order of magnitude of the lateral deflections of the bridge. These deflections are at a maximum at point P1, slightly smaller at point P2 at the midspan, and even smaller at point P3, the approximate position of which is shown using arrows. These photos are from a video shot from a location near to those of the analyzed videos.

These conditions were met in the monitoring of the Kanellopoulos timber bridge (Figure 1), which represents a unique case of a structure with rapidly deteriorating structural characteristics. Experience from people crossing this pedestrian bridge indicated that a few years after its construction in 2000, this bridge became unstable, very sensitive to loading by passengers, and characterized by large lateral deflections (see the video in the Supplementary Material in Stiros and Moschas [9]), reminiscent of the oscillations of the Millennium Bridge, London [10]. For this reason, it was essentially abandoned. Luckily, the overall process of bridge decay has been described by annual monitoring surveys between 2007 and 2016. These surveys aimed to measure the response of the bridge to controlled excitations using geodetic instruments, GNSS (Global Navigation Satellite Systems, i.e., sensors collecting information from GPS and other satellite positioning systems), RTS (robotic theodolites or robotic total stations), and accelerometers. The measurement process and the analysis of the collected data were essentially uniform in all surveys, the details and results of which are summarized in Stiros and Moschas [9], among others. However, results of more recent surveys have not yet been published but they were available for this study; in any case, only minor changes in the structural behavior of the bridge has occurred after 2011/2012 [11]. All surveys were covered by metadata, including video recordings of the experiments by common, low-cost cameras. Preliminary analysis of the video imagery collected during certain surveys indicated that when the damaged bridge was subject to controlled dynamic excitations, the dynamic deflections of some of its parts are clearly visible in short-range video imagery, as is indicated in Figure 2. Since constraints to deflections were known from results of the analysis of geodetic sensors and accelerometers, from published [9,11] and unpublished results, as well as the overall knowledge of the structural behavior of the bridge, the challenge was to examine whether the available video recordings could easily describe the time series of dynamic deflections.

In this article, we describe the methodology we used to estimate the dynamic horizontal deflections of specific points of this bridge using a simple video analysis technique and a low-cost camera to derive some dynamic characteristics of this structure. The basic characteristics of the proposed method were that it focused on significant deflections (amplitude of several mm) and may lead to statistically significant results without any correlation analysis [8]. Additionally, we describe the techniques we used to validate these results, as well as the perspectives and limitations of the proposed methodology.

## 2. The Kanellopoulos Timber Bridge and its Decay

The studied bridge is shown in Figure 1 and is located at the northern entrance of Patras, not far from the Patras University Campus, and was constructed in 2000. It is about 30 m long and 2.9 m wide and is made of Glulam wood and certain metallic elements. The omission of X-bracing below the deck and poor construction of the metal X-bracing at its roof (Figure 2) rendered the structure vulnerable to events producing lateral oscillations. This, in combination with an extraordinary-for-the-area flash icing event and subsequent strong winds flowing normal to the deck direction, maximizing the lateral deflections (cf. Meng et al. [12]), led to a dramatic drop of its first lateral modal frequency in less than two years, and then led to a further, slower drop [11]. However, the vertical stiffness of the bridge was not significantly affected and the bridge remained stiff with a vertical main natural frequency >6 Hz, and hence with millimeter-level deflections at mid-deck. These results come from an analysis of annual surveys of the bridge since 2007. These surveys were based on combinations of various sensors (GNSS, RTS, accelerometers) at various parts of the bridge and were focused on different types of forced excitations. The analysis of oscillations was especially focused on the free attenuation intervals of excitations, which provide evidence of natural frequencies. These results are summarized in various publications, especially in [9,11].

## 3. Methodology

### 3.1. Bridge Quasi-Planar Dynamic Displacements and Impacts in Vision-Based Monitoring

Equation (1) describes the general case of photogrammetric analysis and is typically used to derive displacements from various types of imagery [3,4]. However, in specific cases, the relationship between the displacement and the imagery can be simplified. For example, in the case of a linear displacement on a plane normal to the sightline of the camera, an essentially linear relationship exists between a length in pixels read on an image describing the displacement and the “true” displacement. In this study, we focused on a special case in which displacements were two-dimensional (cf. Pan et al. [8]).

Detailed analysis of measurements of the dynamic displacements in bridges has shown that the deformation along their main (longitudinal) axis is nearly zero [13], and only vertical and lateral displacements are beyond the threshold of identification of most modern instruments. The only exceptions are extremely flexible structures and bridges swaying laterally, not only in their first mode but in higher modes as well, which are rare. For this reason, no displacements along the longitudinal axis of bridges are discussed in the literature. This means that during dynamic movements, the horizontal and vertical deflections are practically confined to a vertical plane that is moving back and forth with time. Lateral shifting of the vertical plane of dynamic deformation can be of the order of a few meters in long suspension bridges [12,14] and up to a few centimeters in common pedestrian bridges up to a few tens of meters long [9,15]. The fact that dynamic deflections are essentially planar in many bridges has important implications in vision-based analysis of their deflections.

If the recording camera is in a stable, known position and orientation, the planar character of the displacements of a monitored bridge will be preserved after any rotation and translation of the coordinate system. This is because rotations correspond to affine transformations, which preserve planarity. Consequently, Equation (1) can be simplified. However, reconstruction of the displacements requires a scale correction. The scale can be computed easily and precisely if the vertical plane containing the deformation vectors is next to, or very close to, horizontal and vertical structural elements of the bridge, as shown in the images analyzed. The ratio of the length of these elements relative to their length in pixels in the image permits deriving scales for correction of the displacement derived from the measurement of the number of pixels in the image. This is of course possible if the amplitude of displacements is small relative to the dimensions of the examined bridge elements, if the distance of the camera from these structural elements is in a range permitting the necessary pixel resolution, and visibility conditions are very good.

### 3.2. Computation of Displacements

For the reasons discussed above, it is possible to compute approximate dynamic displacements with a low-cost camera using the technique explained in the three steps below. This is possible if the study bridge a priori satisfies three criteria: planar dynamic deformation, a significant amplitude of some of its dynamic deflections, and target points next to structural elements permitting scale correction are monitored.

#### 3.2.1. Digitization of the Motion of a Selected Point of the Deforming Bridge

A characteristic moving target point of the bridge that was visible in a series of i=1, 2,…,n images (frames) was identified. Using common software, the variable position of this point relative to each frame shot from a stable camera was translated into a series of coordinates ($x_{vi},\;z_{vi},\;t_{i}),$ where the index *v* indicates video. These coordinates refer to the plane of the image and describe the variations of the location of the target point as a function of time relative to stable (reference) points common in the *n* images (frames).

#### 3.2.2. Scale Correction

In the examined images, certain vertical and horizontal elements of the bridge that were near the control point were marked and their lengths in pixels were measured. The true length of these elements was also measured. A mean quotient of the true length of these elements to their length measured in pixels in the images could then be computed:

(2) $$s_{v}=\ell_{v\;}/p\ell {\;}_{v}\;,{\;s}_{x}=\ell_{x}/p\ell_{x}.$$

The index v corresponds to the vertical axis and the index x to the horizontal axis, and ℓ and pℓ correspond to the length and length in pixels, respectively. The quotients $s_{v}$ and $s_{x}$ define the scales used to transform the coordinates of displacements measured in pixels into scale-corrected, metric displacements ($s_{x}x_{vi},\;0,\;{\;s}_{v}z_{vi},\;t_{i})$. Because this approach is approximate, non-linear scaling effects (lens distortion) were ignored.

#### 3.2.3. Rotation of Images

**Figure 3:**
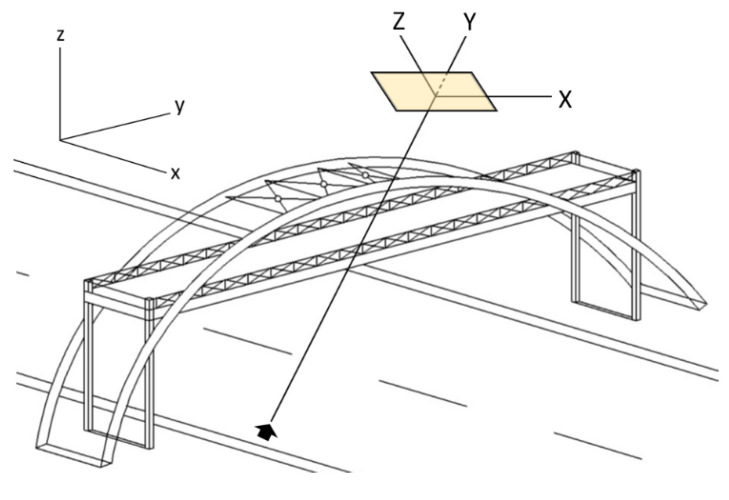
The Cartesian coordinates of the bridge (x, y, z) were transformed into a Cartesian coordinate system (X, Y, Z) defined by the camera image plane (shown as the shaded box). Dynamic displacements along the longitudinal axis *y* of the bridge were minimal, if any, and the dynamic deformation of the bridge was expressed by deflections confined to the x–z plane. For this reason, a simple linear relationship connected the dynamic deflections of the bridge in the x–z plane with the X–Z plane of the camera image. The black arrow indicates the camera.

Since the camera was kept fixed, the video images from which the displacements of the target point were measured were in a plane rotated relative to a bridge plane defined by the vertical and the main longitudinal axes of the bridge (Figure 3). Furthermore, since the coordinates and orientation of the camera and the bridge were known/measured, the translation and rotation angles of the image plane relative to the bridge plane could be easily computed. Hence, an unknown planar displacement of the bridge ${\left[x,\;0,\;z\right]}^{T}$ was mirrored into a displacement ${\left[X,\;0,\;Z\right]}^{T}$ in the image plane. This process is described by Equation (3):

(3) $${\left[X,\;0,\;Z\right]}^{T}={\;R}_{z}R_{x}{\left[x,\;0,\;z\right]}^{T}+T.$$

Matrix T with dimensions 1 × 3 describes the translation of the origin of the two coordinate systems and matrix $R_{z}R_{x}$ is a 3 × 3 matrix reflecting the superimposition of rotations around axes z and x. A detailed expression is given in the Appendix A. Matrix $R_{z}R_{x}$ can be easily inverted because:

(4) $${\left(R_{z}R_{x}\right)}^{-1}={\left[R_{z}R_{x}\right]}^{T}.\;$$

Hence, in ideal conditions (i.e., ignoring errors), from the planar displacement vectors measured in the image plane $\left[X_{i},\;0,{\;Z}_{i}\right]{\;}^{T}$, the planar displacements of the point on the bridge ${\left[x_{i},\;0,{\;z}_{i}\right]}^{T}$ can be computed through a simple linear transformation:

(5) $$\left[x_{i},\;0,{\;z}_{i}\right]{\;}^{T}={\left(\mathrm{RR}\right)}^{T}\;\left[X_{i},\;0,{\;Z}_{i}\right]{\;}^{T}+\;T\;\prime .$$

T′ describes a translation similar to matrix T. However, because various measurements were contaminated by errors, Equation (5) should be replaced by Equation (6):

(6) $${\left[x_{i}^{*},{\;y}_{i}^{*},{\;z}_{i}^{*}\right]}^{T}={\left(\mathrm{RR}\right)}^{T}\;\left[s_{x}x_{vi},\;0,{\;s}_{z}z_{vi}\right]{\;}^{T}+\;T\;\prime ,\;$$

which does not describe a planar deformation. The reason is that ${\left[s_{x}x_{vi},\;0,{\;s}_{z}z_{vi}\right]}^{T}$ and R (as well as T′) contained errors, which were reflected in ${\left[x_{i}^{*},{\;y}_{i}^{*},{\;z}_{i}^{*}\right]}^{T}$ and especially in $y_{i}^{*}\;\neq \;0$. For this reason, the output of Equation (6) should be written as:

(7) $${\left[x_{i}^{*},{\;y}_{i}^{*},{\;z}_{i}^{*}\right]}^{T}={\left[x_{i},\;0,{\;z}_{i}\right]}^{T}+{\left[u_{\mathrm{xi}},{\;u}_{\mathrm{yi}},{\;u}_{\mathrm{zi}}\right]}^{T},$$

with ${\left[u_{\mathrm{xi}},{\;u}_{\mathrm{yi}},{\;u}_{\mathrm{zi}}\right]}^{T}$ describing the errors. The time series of $y_{i}^{*}={\;u}_{\mathrm{yi}}$, which indicates the deviations from a planar deformation, permitted an estimate of the quality of the results:

(8) $$\sigma^{2}=\sum^{\;}{\left(u_{\mathrm{yi}}\right)}^{2}∕n.$$

A preliminary analysis indicated that in the conditions of our experiment, the proposed technique could not provide estimates of deflections below the threshold of 15 mm. For this reason, and since the study bridge remained stiff along the vertical axis despite its damage [16], we focused only on the horizontal deflections, which were expected to be within the resolution limits of the method and the instruments used. Further analysis of the errors is given below.

### 3.3. Computation of Certain Dynamic Characteristics of the Bridge

Each of the videos examined covered a part of a controlled, forced excitation of the bridge, followed by free attenuation intervals. Analysis of the latter permitted computation of the main modal frequency in the lateral axis of the bridge, as well as the damping factor without any formal modal analysis.

## 4. Data

The analyzed videos came from the 2013 and 2015 annual field surveys when the effects of damage were very clear in the bridge, both as structural vicissitudes [11] and as high-amplitude lateral deflections during excitations by pedestrians and wind (Figure 2); video in the Supplementary Materials of Stiros and Moschas [9]). During these field surveys, deflections at the mid-span of the deck were of the order of several centimeters, which was above the identification threshold of 15 mm estimated for the adopted methodology and the video camera used. 

The videos that were selected were parts of two longer videos taken from known points, a few meters away from the SW edge of the bridge, with the camera sighting obliquely to the bridge under excellent visibility conditions. A low-cost but high-resolution pocket camera (16M pixels, 1920 × 1080 resolution) with automatic stability control and common mounting was used. No significant vibrations of the camera due to air currents generated by passing vehicles (cf. Feng and Feng [5]) occurred during the specific experiments. The two videos analyzed in this article covered parts of controlled, forced excitations of the bridge by well-trained parties of students and covered part of a transient and the following free attenuating oscillation (just after the jumping party froze). Details of the process are given in References [13,17,18]. Each of the analyzed videos had a duration of a few seconds and a rate of 30 images per second (frames per second (fps)).

For each of the two measurement years, namely 2013 and 2015, three control points were identified on the south face of the bridge, which were clearly visible in all frames. Point 1 (P1) was near the crown of the bridge, close to its midspan, next to a GPS/GNSS. Point 2 (P2) was at the midspan, on the top of the handrail of the bridge, close to a collocated GPS/GNSS, a reflector of the robotic total station, and an accelerometer. Finally, Point 3 (P3) was at about one-fourth of the span, close to an accelerometer (Figure 2). Near each point, it was possible to identify and measure at least two vertical and two horizontal bridge elements, the edges of which were clearly identified in all video frames such that the scale corrections of Equation (2) were computed. Furthermore, stable reference points were identified in all frames.

In addition to these data, certain qualitative and quantitative constraints of the dynamic behavior of the bridge were available: (1) no significant vertical displacements were expected because the bridge remained stiff in the vertical axis (first natural frequency of the order of 6 Hz despite its decay [16] and (2) lateral deflections of several centimeters at the deck mid-span level were expected with a frequency of the order of 1 Hz [9], where these deflections were attenuated to the deck edges, while the crown of the bridge was characterized by lateral deflections of an amplitude larger than the deck [11]. These are constraints that allowed for evaluation of the performance of the video analysis.

## 5. Computation of Horizontal Deflections

Step 1: Videos were analyzed using the software “DLTdv Digitizing Tool” (biomech.web.unc.edu/dltdv/), which involved locking onto a selected point and certain non-moving reference points each time (Figure 2). The output of each analysis was a CSV file of the type $\left(x_{vi},\;z_{vi},\;t_{i}\right)$, which described the kinematics of the selected point in image pixel units as a function of the time. However, the variation of the vertical coordinate was small and within the error limits.

Step 2: The lengths of certain vertical and horizontal reference lengths, clearly visible in the images, were measured with a tape. The corresponding lengths in pixels in the images were also measured. From these measurements and using Equation (2), the mean scales *s_v_* and *s_x_* were computed for each video. Using these estimates for scale, matrices ($x_{vi},{\;z}_{vi},{\;t}_{i})$ describing the displacement in pixels were transformed into matrices [$s_{x}x_{vi},\;0,{\;s}_{v}z_{vi},{\;t}_{i}]$ describing the corrected metric displacements. The mean scale factors are shown in Table 1.

**Figure 4:**
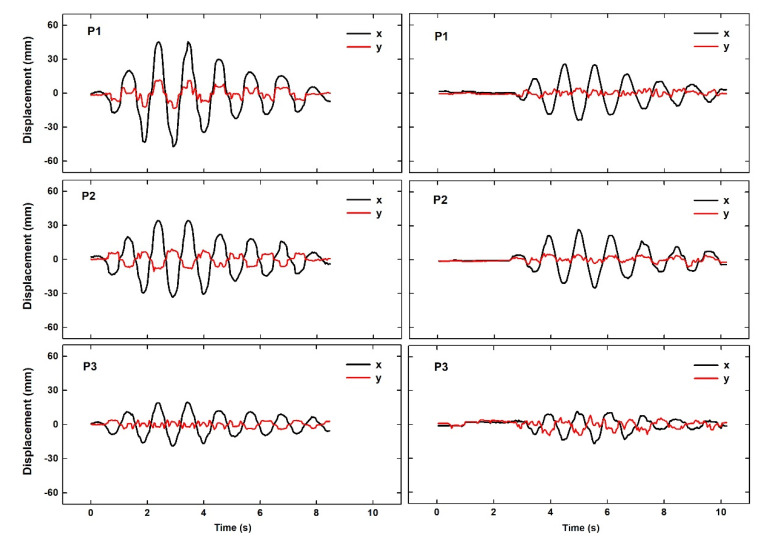
Summary of the computed lateral deflections (axis x) and the spurious longitudinal deflections (axis y) indicating the noise level in the measurements. Differences in the amplitude of displacements in 2013 and 2015 indicate the difference in excitations.

Step 3: Based on survey measurements, the coordinates of the setup point of the camera in each epoch and its pitch (along axis X) and yaw (along axis Z) angle were computed and are summarized in Table 1. These rotation angles were used to compile matrix *R* in Equation (6). The matrix ${\left[x_{i}^{*},{\;y}_{i}^{*},\;0\right]}^{T}$ of deflections in the coordinate system defined by the main axis of the bridge were then computed. The time series of computed deflections of the three control points for the 2013 and 2015 data are plotted in Figure 4. This figure also shows apparent deflections along the longitudinal axis of the bridge, which were negligible, as already explained. The mean square value $\sigma^{2}\;$of these apparent deflections provided an estimate of the order of magnitude of the errors in the specific study, both concerning the method and the type of camera used.

## 6. Structural Characteristics of the Bridge

Because of their design, the analyzed videos covered the transient part of the oscillations and its free attenuation. The latter could directly (i.e., without any formal structural identification analysis) lead to two important characteristics of this bridge: its main modal frequency *f* along the main lateral axis and the damping factor (attenuation coefficient) ζ. f was computed through spectral analysis of displacements x(t), while ζ is computed through a least-squares analysis of Equation (9):

(9) $$x\left(t\right)=\;A\;\sin\left(\omega t\;+\varphi \right)e^{-\zeta \omega t}.$$

**Figure 5:**
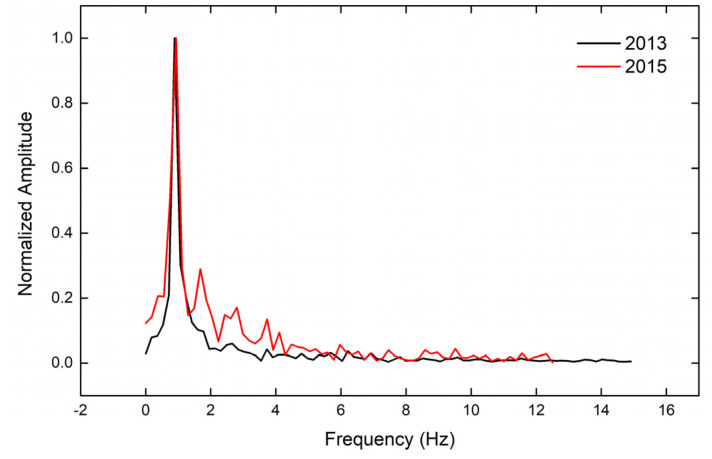
A comparative plot of the spectra of the free attenuation intervals of the oscillation of point P2 for the two years. The spectra are normalized to their maximum amplitude and reflect the first lateral modal frequency of the bridge (only the free attenuation intervals were analyzed).

**Figure 6:**
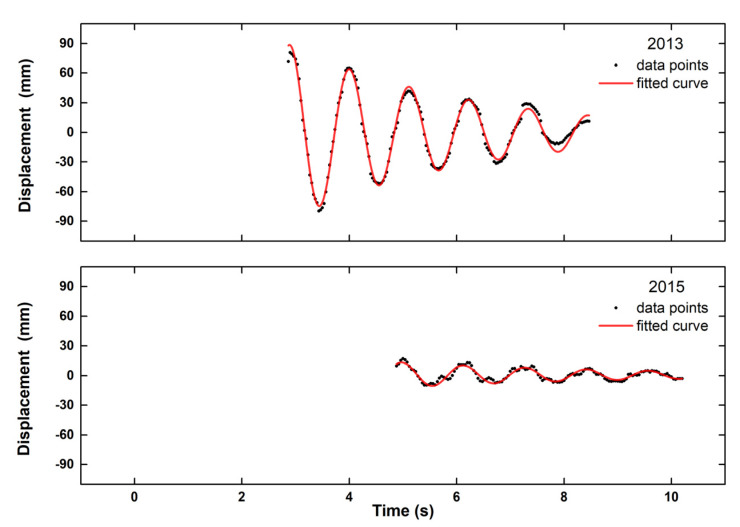
Modeling of the free attenuation intervals of the oscillations of point P2 as free attenuating oscillations based on Equation (9). The period input into the analysis was computed from the spectra of Figure 5.

In this equation, which is valid for ζ<0.1, ω=2πf, φ is the phase characterizing the oscillation, and A is the initial amplitude just before the decay of the motion [19]. Results are summarized in Table 2, and Figure 5 and Figure 6.

## 7. Discussion

Vision-based estimation of deflections of bridges is usually based on high-quality cameras and a rather complicated analysis of digital images [2,20]. In this article, it is shown that under certain conditions, low-cost cameras and a simplified methodology can lead to a time series of displacements. These results first need to be validated and the limitations of the method must be discussed.

### 7.1. Data Validation

The obtained results were first tested for statistical significance, and second for their consistency with structural constraints.

#### 7.1.1. Statistical Significance

Figure 4 and Table 2 indicate that the lateral deflections were larger than the corresponding longitudinal deflections indicating noise. Furthermore, in each case, the corresponding signal-to-noise ratio (SNR, measured as the mean square value of lateral deflections to the mean square value of the apparent longitudinal deflections) indicated statistically significant results for all three points, in both the 2013 and 2015 surveys.

#### 7.1.2. Consistency with Structural Constraints

From a qualitative point of view, as noticed above (Section 4), it was expected that the deflections of P1 were to be higher than those of P2, and those of P2 (mid-span) were to be higher than those of P3. These constraints were satisfied, as the results summarized in Figure 4 and Table 2 indicate.

The previous knowledge of the structural characteristics of the bridge indicated that during excitations, such as those analyzed, the lateral deflections at P2 were expected to reach and even to exceed an amplitude of several centimeters; this was consistent with the results of Table 2 and Figure 4.

The spectral analysis of the free attenuating oscillations led to estimates of the first modal lateral frequency that were very similar in all points for each epoch, and the values were consistent with values derived from independent measurements (Table 3). Damping factors computed for the three points were also consistent with each other (Table 2) and of an order of magnitude expected in a timber bridge with high deflections (cf. Bachmann et al. [19], p. 4). What is especially impressive is that the recorded deflections were characterized by a pattern that is expected in free attenuating oscillations, even for points with a low SNR (as evident from the difference between the red and black curves (Figure 4).

These results provide evidence that this simplified video analysis led to reliable data.

### 7.2. Possible Structural Implications for the Kanellopoulos Bridge

The results of the video analysis indicate a minimal reduction of the stiffness of the bridge between 2013 and 2015 (the first modal frequency changed from 0.90 to 0.87 Hz). This difference was consistent with the overall trend of the bridge that was derived from an analysis of a survey and accelerometer data and summarized in Table 3, as well as with eye-witness evidence for a gradual structural decay [11]. However, such evidence comes from only two experiments, the results are within uncertainty limits, and their documentation requires a formal statistical analysis (cf. Stiros and Moschas [21]).

### 7.3. Limitations and Applications of the Method

The method proposed is simple and may lead to significant results, as has been shown above. However, it is an approximate method and it can only be applied under certain conditions. The first condition is that it is only efficient for slender structures if deflections are above a certain threshold. This threshold depends on the resolution of the camera and its distance from the observed structure such that displacements are reflected as a signal of several pixels. In the case of a low-cost camera, as was used during this study, and in good visibility conditions, the method is suitable for deflections above the threshold of 10–15 mm and at distances of up to a few tens of meters; these conditions are not unusual, even for hardware with high specifications [2,20]. Another requirement of the proposed method is that the morphology of the analyzed bridge and its background should permit a stable reference frame for the video and that the analyzed bridge should contain elements that are next to control points and are seen in all video frames such that scale corrections are possible.

## 8. Conclusions

It was shown that the analysis of low-cost video images using a simple approximate technique permits the reconstruction of deflections of flexible bridges and even the computation of some of their structural characteristics. This result is possible under certain conditions: the deformation is two-dimensional, deflections of the selected target points are characterized by a signal exceeding the pixel resolution, the camera is in a fixed position and orientation, and finally, the video image covers stable points defining a stable reference system and structural elements near the selected target points such that the scale of the photo in the two examined axes can be defined. This approach was developed for dynamic displacements but it can also be applied in the case of semi-static and static motions (for example, static loading tests). In this last case, the resolution and accuracy of the method are expected to be higher (and hence the method may be applied to stiffer structures) because the noise in images produced by atmospheric turbulence, a major threat for optical techniques around bridges, is randomized and minimized (cf. Stiros and Moschas [21]).

The proposed method is certainly not limited to certain types of bridges since it may also be applied to various other structures, both in close range in the laboratory and with a longer range in the field. For example, it can be applied in laboratory tests for control of the deformation of various structural elements, avoiding expensive contact methods. It can also be applied in various types of structures in the field, as long as they are subject to 2-D deformation. However, significant results are expected if the whole series of images show stable objects marking a stable reference frame. Furthermore, since a single video time series of displacement allows for more than one point to be measured, the rotation angles (e.g., pitch, yaw, etc.) of rigid bodies can also be computed.

## Figures and Tables

**Table 1:** Data used for the image coordinate transformation.

Parameter	2013			2015		
	P1	P2	P3	P1	P2	P3
**Scale factor s_x_** **(pixels/mm)**	21.55	21.45	13.43	6.39	6.39	3.01
**Scale factor s_v_** **(pixels/mm)**	41.13	40.94	24.15	17.03	17.03	6.10
**Camera rotation angle** **pitch a (°)**	29.7			40.3		
**Camera rotation angle** **yaw c (°)**	40.8			40.0		

**Table 2:** Summary of the results.

Parameter	2013			2015		
	P1	P2	P3	P1	P2	P3
**Max deflection x** **(amplitude) (mm)**	47	34	19	26	26	13
**Max apparent deflection y** **(amplitude) (mm)**	25	17	15	5	7	10
**xi2n**	410	256	81	100	102	31
**yi2n**	25	21	05	3	5	12
**SNR**	16	12	16	33	20	3
**f (video)**	0.90	0.90	0.90	0.87	0.87	0.86
**f (GNSS/RTS)**		0.92/0.93			0.88	
**ζ (%) (video)**	5.4	4.9	3.9	5.5	4.9	5.5

SNR: Signal-to-noise ratio, GNSS: Global Navigation Satellite Systems, RTS: Robotic total station.

**Table 3:** Variations of the first lateral natural frequency f of the Kanellopoulos Bridge, known from the analysis of geodetic sensors and accelerometers (from Thalla and Stiros [11], with additions) and those derived in this study.

Year	f (Known)	f (Video)
2007	2.63	
2009	1.02	
2010	0.99	
2012	0.95	
2013	0.92–0.93	0.90
2015	0.88	0.87

## Appendix A

Equation (1) in its full form is:

(A1) $$\left[\;\begin{array}{c} x_{p}\\ y_{p}\\ 1 \end{array}\;\right]=k\left[\begin{array}{ccc} f_{o} & \gamma & x_{o}\\ 0 & f_{y} & y_{o}\\ 0 & 0 & 1 \end{array}\right]\;\left[\begin{array}{cc} r_{11} & r_{21}\\ r_{12} & r_{22}\\ r_{13} & r_{23} \end{array}\;\begin{array}{cc} r_{13} & t_{x}\\ r_{23} & t_{y}\\ r_{33} & t_{z} \end{array}\right]\;\left[\begin{array}{c} x_{r}\\ y_{r}\\ \begin{array}{c} z_{r}\\ 1 \end{array} \end{array}\right]=\;\mathrm{kAR}\left[\begin{array}{c} x_{r}\\ y_{r}\\ \begin{array}{c} z_{r}\\ 1 \end{array} \end{array}\right],$$

where index p indicates the image (photo) and r indicates the reference (“world” or “global”) coordinates, k is a scale factor, A is a matrix expressing the deformation of the image through the camera, and R is a matrix expressing the rotation and translation of the camera [3,4].

The full form of the matrix product R_z_R_x_ in Equation (3) is:

(A2) $$\left[\begin{array}{ccc} \cos\;c & -\sin\;c & 0\\ \sin\;c & \cos\;c & 0\\ 0 & 0 & 1\; \end{array}\right]\left[\begin{array}{ccc} 1 & 0 & 0\\ 0 & \cos\;a & -\sin\;a\\ 0 & \sin\;a & \cos\;a \end{array}\right]=\left[\begin{array}{ccc} \cos\;c & -\cos\;a\;\sin\;c\; & \;\sin\;a\;\sin\;c\\ \sin\;c & \;\;\;\cos\;a\;\cos\;c\; & -\cos\;c\;\sin\;a\\ 0 & \sin\;a & \cos\;a \end{array}\right],$$

where c is the angle of rotation along axis z and a is the angle of rotation along axis x, as shown in Table 1.
